# *Trypanosoma cruzi* MSH2: Functional analyses on different parasite strains provide evidences for a role on the oxidative stress response^☆^

**DOI:** 10.1016/j.molbiopara.2010.11.001

**Published:** 2011-03

**Authors:** Priscila C. Campos, Viviane G. Silva, Carolina Furtado, Alice Machado-Silva, Wanderson D. DaRocha, Eduardo F. Peloso, Fernanda R. Gadelha, Marisa H.G. Medeiros, Gustavo de Carvalho Lana, Ying Chen, Rebecca L. Barnes, Danielle Gomes Passos-Silva, Richard McCulloch, Carlos Renato Machado, Santuza M.R. Teixeira

**Affiliations:** a Departamento de Bioquímica e Imunologia, Instituto de Ciências Biológicas, Universidade Federal de Minas Gerais, Belo Horizonte, MG, Brazil; b Departamento de Bioquímica, Instituto de Biologia, Universidade Estadual de Campinas, Campinas, SP, Brazil; c Departamento de Bioquímica, Instituto de Química, Universidade de São Paulo, SP, MG, Brazil; d Departamento de Estatística, Instituto de Ciências Exatas, Universidade Federal de Minas Gerais, Brazil; e Wellcome Centre for Molecular Parasitology, University of Glasgow, Glasgow, UK

**Keywords:** *Trypanosoma cruzi*, DNA repair, MSH2, Oxidative stress, *Trypanosoma brucei*

## Abstract

Components of the DNA mismatch repair (MMR) pathway are major players in processes known to generate genetic diversity, such as mutagenesis and DNA recombination. *Trypanosoma cruzi*, the protozoan parasite that causes Chagas disease has a highly heterogeneous population, composed of a pool of strains with distinct characteristics. Studies with a number of molecular markers identified up to six groups in the *T. cruzi* population, which showed distinct levels of genetic variability. To investigate the molecular basis for such differences, we analyzed the *T. cruzi* MSH2 gene, which encodes a key component of MMR, and showed the existence of distinct isoforms of this protein. Here we compared cell survival rates after exposure to genotoxic agents and levels of oxidative stress-induced DNA in different parasite strains. Analyses of *msh2* mutants in both *T. cruzi* and *T. brucei* were also used to investigate the role of *Tcmsh2* in the response to various DNA damaging agents. The results suggest that the distinct MSH2 isoforms have differences in their activity. More importantly, they also indicate that, in addition to its role in MMR, TcMSH2 acts in the parasite response to oxidative stress through a novel mitochondrial function that may be conserved in *T. brucei*.

## Graphical abstract

**Graphical abstract:**

*T. cruzi* II strains accumulate more 8-oxoguanine in the kDNA after hydrogen peroxide-induced 18 oxidative stress than *T. cruzi* I strains. NT: untreated; T: treated.

**Figure:**
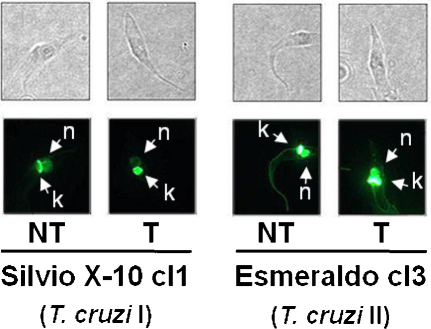

**Research highlights:**

▶ Distinct levels of DNA mismatch repair activity are found among *T. cruzi* strains. ▶ In *T. cruzi* and *T. brucei*, MSH2 has a mitochondrial function involved in the response to oxidative stress.

## 1 Introduction

*Trypanosoma cruzi* is a protozoan parasite of great medical importance, since it causes Chagas’ disease (or American Trypanosomiasis), a malady endemic throughout Latin America, with an estimated 50,000 deaths/year and 100 million people at risk (http://who.int/tdr/diseases/Chagas). *T. cruzi* belongs to the order Kinetoplastida, which is characterized by the presence of one flagellum and a single mitochondrion in which is situated the kinetoplast, a specialized DNA-containing organelle. Though separated by many millions of years of evolution, *T. cruzi* is related to another kinetoplastid parasite, *T. brucei* [1], which continues to afflict the economy and health of the people of sub-Saharan Africa.

*T. cruzi* has a heterogeneous population composed by a pool of strains that circulate in the domestic and sylvatic cycles involving humans, insect vectors and animal reservoirs. Based on studies with a number of molecular markers, the taxon was divided initially in two well-defined groups, so-called *T. cruzi* I and *T. cruzi* II lineages [2]. More recent studies proposed the existence of six discrete typing units named *T. cruzi* I to VI [3]. Although the *T. cruzi* population is predominantly clonal, a few hybrid lineages have been identified, including the clone CL Brener, chosen as the reference strain for the genome project [4].

Several reports highlighted the differential genetic diversity between *T. cruzi* I and II lineages and, more importantly, the intra-group variability observed within *T. cruzi* II. Phylogeny studies of DHFR-TS and TR sequences from 31 strains showed that all sequences from *T. cruzi* I strains are monophyletic, whereas sequences from *T. cruzi* II strains are paraphyletic and fall into 3 clades [5]. Analysis of *T. cruzi* multi-gene families showed that paralogous sequences encoding amastin, an amastigote surface glycoprotein, and TcAG48, an antigenic RNA binding protein, present higher intragenomic variability in *T. cruzi* II and CL Brener strains, when compared with *T. cruzi* I strains [6]. Likewise, whereas sequences encoding the Tc52 immuno-regulatory protein are homogeneous in strains belonging to the *T. cruzi* I lineage, they show higher sequence polymorphisms in *T. cruzi* II strains [7].

Components of DNA repair pathways are key players in the processes that result in changes in genetic variability within any cell population. Germline mutations in DNA mismatch repair (MMR) genes are associated with susceptibility to hereditary nonpolyposis colorectal human cancer (HNPCC) [8]. Mutator populations of bacteria, yeast and mammalian cells with defects in mismatch repair have also been described in isolates showing increased frequency of drug resistance [9–11]. Post-replicative DNA mismatch repair promotes genetic stability by repairing DNA replication errors, inhibiting recombination between non-identical DNA sequences and participating in responses to DNA damage induced by genotoxic agents, such as *N*-methyl-*N*′-nitro-*N*-nitrosoguanidine (MNNG) and cisplatin [12,13]. MMR components are also involved in the response to oxidative lesions in DNA such as 7,8-dihydro-8-oxoguanine (8oxoG) [14]. The initial steps of MMR are accomplished by heterodimers of MSH proteins, named MSH1 to MSH7 in eukaryotes. In *Saccharomyces cerevisiae,* the MSH2/MSH6 complex, or MutSα, recognizes nuclear base–base mismatches and small (1–2 bp) insertion–deletion (InDel) loops, while MSH2/MSH3 heterodimers, or MutSβ, recognizes a range of small and larger loop-outs. *S. cerevisiae* MSH1 protein is involved in mitochondrial genome stability [15].

TcMSH2 was the first component of the MMR machinery described in *T. cruzi* [16]. Analyses of the *TcMSH2* single copy gene present in different strains of the parasite demonstrated the existence of three isoforms of this protein, named MSH2A, B and C, which correlate with the division in three phylogenetic *T. cruzi* lineages [17]. A role of MMR in creating differences in genetic variability among *T. cruzi* strains was initially unveiled by studies showing that strains representative of *T. cruzi* II displayed allelic variation of nuclear microsatellite *loci* induced by hydrogen peroxide, the same not occurring in a strain belonging to the *T. cruzi* I lineage [18]. Moreover, parasites from MSH2 haplogroups B and C were more resistant to cisplatin treatment, as previously reported in other MMR-deficient cells [13]. These studies suggest that, at least under genotoxic stress conditions, strains belonging to the *T. cruzi* I lineage (presenting the A isoform of *TcMSH2* gene) have a more efficient MMR activity than *T. cruzi* II strains [18]. They also support the hypothesis that the distinct genetic diversity found in the two *T. cruzi* lineages might be consequence of different levels of MMR efficiency. Therefore, to better understand the mechanisms involved in generating genetic diversity in *T. cruzi* and, more precisely, the role of the MMR pathway, we investigated the response of DNA damaging agents in strains representative of the two main lineages and the activities of different isoforms of *T. cruzi* MSH2 proteins. Using gene deletion analysis to examine the response to oxidative damage, we uncovered a novel function for MSH2, related to mitochondrial DNA repair, which is conserved in both *T. cruzi* and *T. brucei*.

## 2 Materials and methods

### 2.1 Strains and growth conditions

Five cloned *T. cruzi* strains (Col1.7G2, Silvio X-10 cl1, Esmeraldo cl3, JG and CL Brener) were used. Genotyping of each strain using several markers demonstrated that Col1.7G2, Silvio X-10cl1 belong to *T. cruzi* I lineage whereas Esmeraldo cl3 and JG are representatives of *T. cruzi* II lineage [19]. CL Brener is a hybrid strain (*T. cruzi* II/III), reference for the genome sequencing project [4] and it was selected for the knockout experiments. Epimastigotes were maintained as described [20]. *T. brucei* wild type and *MSH2* deletion mutants were grown as previously described [21].

### 2.2 TcMSH2 amino acid sequence analysis and northern blot assays

*TcMSH2* sequences from CL Brener were retrieved from GeneDB (accession numbers Tc00.1047053509643.80 and Tc00.1047053507711.320), whereas the Col1.7G2 sequence was obtained from a cloned PCR amplified fragment as described [22]. Deduced amino acid sequences were aligned using ClustalW version 1.8 and all protein sequences were run against the Pfam [23], Prosite [24] and InterPro [25] databases. For Northern blot analysis, total RNA (25 μg) was separated on formaldehyde agarose gels, blotted onto nylon membranes, cross-linked through UV irradiation and probed with [α-^32^P] labeled *TcMSH2* as previously described [19]. The probe was labeled according to the Megaprime DNA labeling (GE Healthcare) protocol and the signals were quantified using the ImageJ program (http://rsbweb.nih.gov/ij/).

### 2.3 Treatment of the strains with genotoxic agents

Cultures with 10^7^ parasites per mL were incubated in 24-well plates with different concentrations of MNNG (*N*-methyl-*N*′-nitro-*N*-nitrosoguanidine) (provided by Dr. Álvaro Augusto da Costa Leitão, Instituto de Biofísica Carlos Chagas Filho, RJ), cisplatin (Quiral Química do Brasil S/A), or H_2_O_2_ (Merck), in the presence or absence of 3 μM of the MMR inhibitor cadmium chloride, as indicated. Previous studies have shown a wide range for the H_2_O_2_ IC_50_ using various *T. cruzi* strains, varying from 98 to 190 μM [26,27]. After incubation for 3 or 5 days, cell densities were measured with a haematocytometer using Erythrosin B exclusion.

### 2.4 Measurement of 8-oxoguanine accumulation

Two different protocols were used to assess the 8-oxoG accumulation in *T. cruzi* DNA strains. In the *in situ* experiment, a protocol adapted from Struthers et al. [28] was used. Epimastigotes were incubated in the presence of 200 or 300 μM hydrogen peroxide for 20 min at 28 °C, washed twice with PBS and fixed with 4% paraformaldehyde. Aliquots (20 μL) of the cell suspension were distributed into wells of 8-wells chambered-slides. After 1 h of incubation at 4 °C, cells were permeabilized with 0.2% Triton X-100, treated with 100 μg/mL RNase A and incubated with FITC-conjugated avidin (5 μg/mL final concentration) for 1 h at room temperature in the absence of light*.* After washing with PBS and mounted with a solution of 9:1 Glicerol:Tris–HCl, pH 9.0, the slides were visualized under a fluorescence microscope in a 100× oil immersion. Pre-incubation of FITC-conjugated avidin with 0.5 mM of 8oxodG results in 80% decrease in the fluorescence signal, whereas pre-incubation with dGTP has no significant effect on parasite labeling. Fluorescence intensities were averaged with the ImageJ program and plotted as fluorescence arbitrary units (average fluorescence intensity measured in 100 cells after subtracting the average background intensity). Background signals were measured in 100 fields, randomly chosen on the slides.

The 8-oxoG accumulation was also assessed by HPLC-electrochemical detection. Cells (10^9^/mL) were treated with 20 mM H_2_O_2_ for 1 h at 28 °C, washed with PBS, and the DNA was isolated by the chaotropic NaI method [29] in the presence of 0.1 mM desferroxamine. DNA samples (100 μg) were treated with nuclease P_1_ and alkaline phosphatase and analyzed by HPLC. Samples (100 μg) of digested DNA were injected into the HPLC/electrochemical detection system consisting of a Shimadzu model LC-10AD pump connected to a Luna C_18_ (Phenomenex, Torrance, CA, USA) reverse-phase column (250 mm × 4.6 mm ID, particle size 5 μm). The flow rate of the isocratic eluent (50 mM potassium phosphate buffer, pH 5.5, and 8% methanol) was 1 mL/min. Coulometric detection was obtained with a Coulochem II detector (ESA, Chemsford, MA, USA). The potentials of the two electrodes were set at 120 and 280 mV. Elution of unmodified nucleosides was monitored simultaneously with a Shimadzu SPD-10A UV detector set at 254 nm. A Shimadzu Class-LC10 1.6 software was used to calculate the peak areas. The molar ratio of 8-oxodG to dG in each DNA sample was determined based on coulometric detection at 280 mV for 8-oxodG and on absorbance at 254 nm for dG in each injection.

### 2.5 Analysis of nuclear and mitochondrial genomes in T. brucei

*T. brucei* cultures were treated with H_2_O_2_ as described previously [22]. Paraformaldehyde-fixed cells were spotted onto microscope slides mounted with vectashield containing DAPI (4,6-diamidino-2-phenylindole) (Vector Laboratories Inc.). DNA configurations of the cells were analyzed, blind, by 2 researchers independently, and >500 cells were counted for each data point*.* Microscopic analysis was performed using an Axioskop 2 microscope (Zeiss) and images obtained using Openlab software (Improvision).

### 2.6 Analysis of oxidative metabolism

Epimastigotes were incubated for 18 h at 28 °C in a 96-well plate in the presence of 20 μL of 3-(4,5-dimethylthiazol-2-yl)-2,5-diphenyltetrazolium bromide (MTT) (5.0 mg/mL MTT in PBS pH 7.6 as stock solution) and the final volume adjusted to 500 μL with culture medium. The reaction was stopped by the addition of 1.0 mL 0.04 N hydrochloric acid in 2-propanol. After 30 min, the lysate was vigorously mixed until completely homogenized. The supernatant was separated by centrifugation at 200 × *g* for 10 min and its absorbance was measured at 570 nm. NADPH production by the pentose phosphate pathway was measured as previously described [27].

### 2.7 Plasmid constructions and parasite transfections

The full-length *TcMSH2* gene was amplified from genomic DNA (CL Brener strain). The PCR was performed using primers forward and reverse carrying XbaI and NotI restriction sites, respectively, and amplicons were inserted into TOPO pCR4 (Invitrogen) generating the plasmid TOPO*TcMSH2*. Fragments containing the resistance genes hygromycin phosphotransferase (HYG) or neomycin phosphotransferase (NEO) were obtained after digestion of the pROCK-GFP-HYG or pROCK-GFP-NEO [30] with XhoI/NheI and ligated to TOPO*TcMSH2*, digested with the same enzymes. The resultant plasmids presenting each one of the resistance genes flanked by N and C-terminal fragments of *TcMSH2* coding sequence were used to delete the *TcMSH2* alleles by homologous recombination. Before the transfection, both plasmids (TOPO N-Neo-C and TOPO N-Hyg-C) were digested with XbaI and NotI and the respective 1.7 Kb and 1.9 Kb fragments were gel-purified. Approximately 50 μg of gel-purified fragments was used to transfect epimastigotes by electroporation according to DaRocha et al. [30]. In order to generate *TcMSH2* knockouts, fragments containing 5′and 3′ sequences of different alleles were also generated by gene SOEing [31]. *TcMSH2* upstream and downstream regions (approximately 500 pb each) were PCR-amplified and fused flanking a PCR-generated cassette containing the *HYG* gene. Transfection of epimastigotes was performed by electroporation with 50 μg DNA as described previously [30]. Twenty-four hours after transfection, 250 μg/mL of Hygromycin B or G418 was added to the cultures and selected population was obtained approximately 30 days after transfection. Clones were obtained by limiting dilution or by plating on semi-solid agar plates, after 30 days of incubation at 28 °C.

### 2.8 Recombinant protein expression

The sub-fragment encoding the N-terminal region (amino acids 1–442) of the TcMSH2 protein was obtained from digestion of the plasmid TOPO2.1 containing the *TcMSH2* gene and cloned directionally into the pET21a+ vector (Novagen), to generate a truncated His-tagged recombinant protein TcMSH2(t). The partially purified recombinant protein obtained from isopropylthiogalactoside induced bacterial cultures was electrophoresed on a 10% SDS-polyacrylamide gel. The 48 kDa corresponding band was excised and used to raise polyclonal antibodies in BALB/c mice by subcutaneous inoculation with Freund's adjuvant*.* Animals were boosted twice at 2 weeks intervals and bled after 6 weeks.

We used a MBP (maltose binding protein) gene fusion system to sub-clone and express a full-length *TcMSH2B* gene, PCR-amplified from genomic DNA using the following primers flanked by EcoRI and XbaI restriction sites, respectively. The amplicon was inserted into TOPO pCR4, which was digested with EcoRI and XbaI and the fragment was inserted into pMal-c2G (New England Biolabs). The recombinant protein obtained from isopropylthiogalactoside-induced bacterial cultures at 28 °C was purified by affinity chromatography on amylose columns.

### 2.9 ATPase assays

ATPase assays were carried out as described [32], using 2 pmol of purified MSH2::MBP or MBP alone (total volume 20 μL). Aliquots (2 μL) were removed at different time intervals (0 and 2 h) and the reactions were terminated by the addition of an equal volume of formamide. Samples were kept on ice before separation on a 20% polyacrylamide gel. Gels were exposed to a photographic film for 1 h at room temperature and the bands corresponding to ATP were quantified using a Phosphoimager Typhoon 8600 (Amersham). To test the effect of cadmium chloride on the ATPase activity, the reagent was added to a final concentration of 10 μM.

### 2.10 Statistical analyses

The results shown in this work were from triplicate determinations and represent three independent experiments performed by identical methods. The unpaired *t* test was used to determine the statistical significance (*p* value) of differences at each pair of groups. All analyses were performed using a *p* value of 0.05 with independent samples with unknown and different variances. For multiple comparisons, the one-way analysis of variance (ANOVA) was used, followed by Tukey's test.

## 3 Results

### 3.1 *T. cruzi* strains display differences in the susceptibility to alkylating agents and hydrogen peroxide

**Supplementary Fig. 1:**
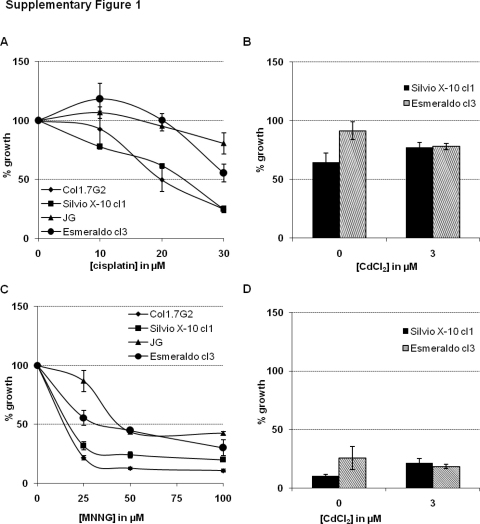
Effect of alkylating agents on the growth of *Trypanosoma cruzi* strains in the presence or absence of cadmium. Exponential cultures of epimastigotes from different *T. cruzi* strains were grown in LIT media containing (A) 10, 20 and 30 μM cisplatin; (B) 30 μM cisplatin in the presence or absence of 3 μM Cd^2+^; (C) 25, 50 and 100 μM MNNG or (D) 100 μM MNNG in the presence or absence of 3 μM Cd^2+^. Viable cell numbers were determined after 3 days (for MNNG treatment) or 5 days (for cisplatin treatment) with erythrosine (B). Results are shown as mean ± SD of 3 independent experiments performed in triplicate.

Cisplatin, a drug widely used in the treatment of cancer, forms intra and inter-strand Platinum-DNA cross-links, which kill the cell if inadequately repaired. *N*-methyl-*N*′-nitro-*N*-nitrosoguanidine (MNNG) is an alkylating agent that induces methylation in the O^6^ position of guanine [13]. MMR-proficient cells are more susceptible to both drugs since MMR proteins may act as DNA damage signal transducers to downstream effectors, participating in the initiation of cell death [33]. We investigated the effect of cisplatin and MNNG on different *T. cruzi* strains by incubation with increasing drug concentrations and determining cell viability after 5 days. The two strains that belong to the *T. cruzi* I group, Silvio X-10 cl1 and Col1.7G2, were more sensitive to treatment with both drug when compared to Esmeraldo cl3 and JG strains (which are *T. cruzi* II strains). The results shown in Supplementary Fig. 1A and C extend our previous observations [18] to a wider range of strains, confirming that there is a clear distinction between strains from *T. cruzi* I and II groups regarding the response to both DNA damaging agents. At cisplatin concentrations of 20 and 30 μM and MNNG concentrations of 25 and 50 μM, the differences between *T. cruzi* I and *T. cruzi* II strains are statistically significant (*p* ≤ 0.05). The mechanisms underlying the difference in the sensitivity to these drugs involve MMR, since, when we repeated the treatment in the presence of cadmium, a potent inhibitor of ATP binding and hydrolysis activities of MSH2-MSH6 complex [34], the differences previously observed between the strains were abolished (Supplementary Fig. 1B and D).

**Supplementary Fig. 2:**
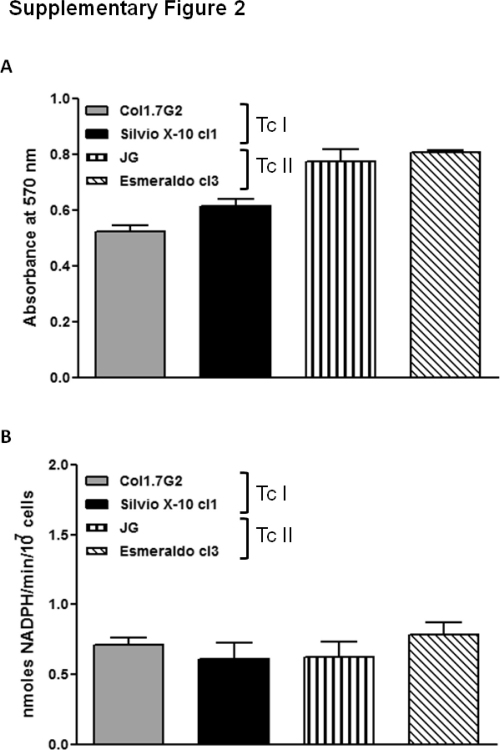
Comparative analysis of the oxidative metabolism among *T. cruzi* strains. In A are shown the levels of MTT reduction ability in different *T. cruzi* strains. Cultures in log phase were incubated with MTT at 28 °C for 18 h, lysed with isopropanol:HCl (299:1) and the absorbance was assessed at 570 nm. In B is shown the NADPH production by the pentose phosphate pathway determined in different strains.

**Fig. 1:**
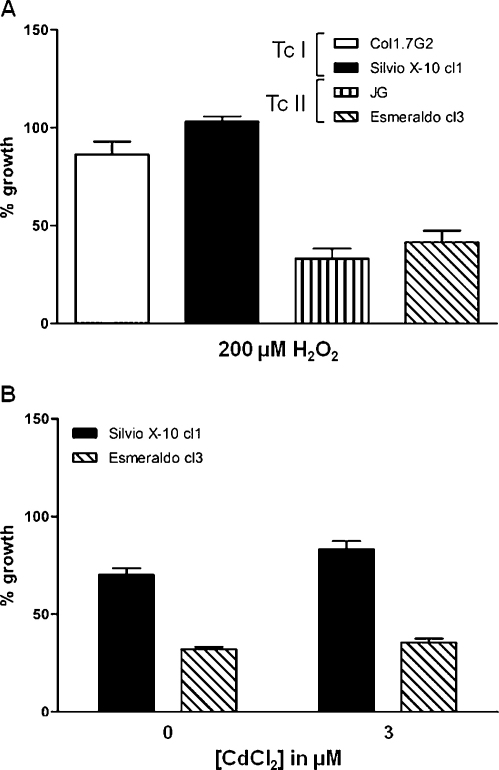
Effect of H_2_O_2_-induced oxidative stress on the growth of *Trypanosoma cruzi* strains in the presence or absence of cadmium. Exponential cultures of epimastigotes were grown in LIT media containing (A) 200 μM H_2_O_2_ or (B) 200 μM H_2_O_2_ in the presence or absence of 3 μM Cd^2+^ for 5 days (B). Viable cells were determined with erythrosine B. Results are shown as mean ± SD of three independent experiments performed in triplicate.

Oxidative DNA damage, which can arise from endogenous metabolism, represents a particularly significant threat for an intracellular parasite. To further compare the response of *T. cruzi* I and II parasites to genotoxic agents, we investigated the ability of different *T. cruzi* strains to proliferate in the presence of hydrogen peroxide (H_2_O_2_). Base lesions arising from oxidative DNA damage, such as 8-oxoG, if not removed by base-excision repair (BER), are recognized by MMR [14]. To test the response of *T. cruzi* strains to oxidative damage, 200 μM of H_2_O_2_ was added to epimastigote cultures and cell viability was determined after 5 days. As shown in Fig. 1A, the two groups of strains exhibited distinct responses to H_2_O_2_ (*p* ≤ 0.05). In contrast to the results observed with cisplatin and MNNG treatments, *T. cruzi* I strains (Col1.7G2 and Silvio X-10 cl1) were more resistant to hydrogen peroxide treatment than *T. cruzi* II strains (JG and Esmeraldo cl3). These differences cannot be attributed to differences in the redox potential of the strains analyzed, as indicated by the results of comparative analysis of the oxidative metabolism of the strains using the MTT reduction assay (Supplementary Fig. 2A) and measuring NADPH production (Supplementary Fig. 2B). Also, in contrast to the response to cisplatin and MNNG treatments, cadmium did not abolish the differential responses of the *T. cruzi* strains to H_2_O_2_. As shown in Fig. 1B, when parasites were incubated with hydrogen peroxide in the presence of this MMR inhibitor, survival rates of the Silvio X-10 (*T. cruzi* I strain) remain higher than the Esmeraldo strain (*T. cruzi* II) (*p* ≤ 0.05).

**Supplementary Fig. 3:**
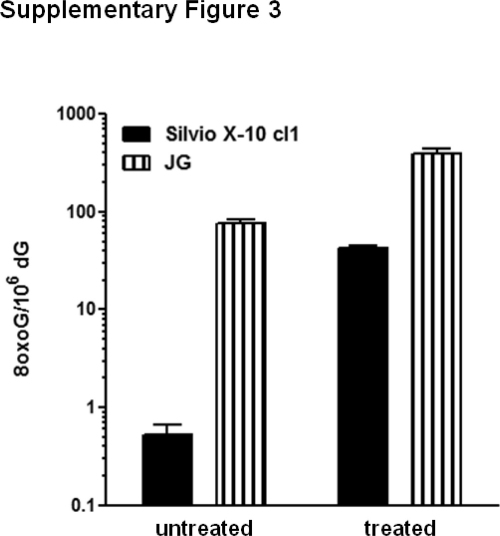
DNA levels of 8-oxoguanine (8-oxoG) among different *Trypanosoma cruzi* strains measured by HPLC. Parasites (10^9^/mL) were treated with 20 mM H_2_O_2_ for 1 h and the purified genomic DNA was analyzed for 8-oxoG content by HPLC as described in Section 2. The data represent the mean ± SD of three independent experiments.

**Fig. 2:**
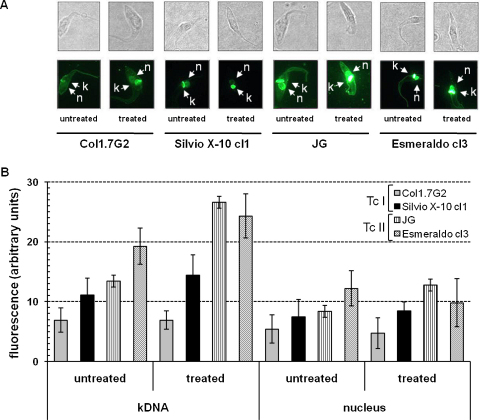
Accumulation of 8-oxoguanine in different *T. cruzi* strains. Exponential cultures of epimastigotes (early log phase) were treated with 200 μM H_2_O_2_ for 20 min and fixed with 4% paraformaldehyde. After transferred to chamber glass slides they were incubated with 5 μg/mL avidin-FITC for 1 h. Slides were visualized in a bright field or by fluorescence (A) in a 100× oil immersion and fluorescence intensity was averaged with the ImageJ program (http://rsbweb.nih.gov/ij/) and plotted as fluorescence arbitrary units, as shown in B. The positions of nucleus (n) and the kinetoplast (k) are shown. Each bar represents the mean ± SD of 100 cells analyzed.

To assess more directly the effect of H_2_O_2_, we examined the accumulation of 8-oxoG in the genome of different *T. cruzi* strains before and after H_2_O_2_ treatment using avidin-conjugated FITC. Avidin is shown to bind with high specificity to 8-oxoG and has been used to detect oxidative DNA damage in different cell types [28]. As shown in Fig. 2A, strains belonging to *T. cruzi* I lineage (Col1.7G2 and Silvio X-10 cl1) present lower levels of the oxidized base than *T. cruzi* II strains (JG and Esmeraldo cl3). This difference (*p* ≤ 0.05) is due to an increased accumulation of 8-oxoG in the kDNA of *T. cruzi* II strains after 20 min in the presence of 200 μM H_2_O_2_ (Fig. 2B). An increased accumulation of 8-oxoG in the DNA of a *T. cruzi* II strain (*T. cruzi* II) compared to a *T. cruzi* I (Silvio X-10) was also observed in experiments using HPLC/electrochemical detection analysis. Because of its high sensitivity and low saturation values, this method detected a much higher difference (145-fold) in 8-oxoG levels (Supplementary Fig. 3).

**Supplementary Fig. 4:**
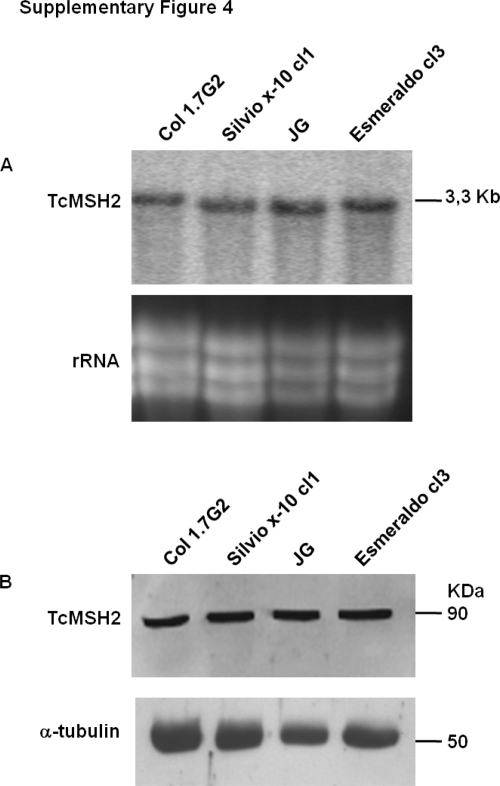
TcMSH2 levels among different *T. cruzi* strains. In A: Northern blot analysis. Twenty-five micrograms of total RNA extracted from epimastigotes of Col1.7G2, Silvio X-10 cl1, JG and Esmeraldo cl3 were hybridized with the same radiolabeled probe indicated in Supplementary Fig. 6E. The bottom part of the figure shows ethidium bromide staining of rRNA bands to indicate the amount of RNA loaded on the gel. In B: Western blot analysis. Protein extracts were separated by SDS-PAGE and then transferred to nitrocellulose filters, and TcMSH2 were detected using polyclonal antibodies anti recombinant TcMSH2.

To verify whether the differences in the response to DNA damaging drugs could be due to differences in the expression of MSH2 among strains, we performed northern and Western blot analyses*.* As shown in Supplementary Fig. 4, no obvious differences the expression of *TcMSH2* mRNA or protein in all strains analyzed were found.

### 3.2 TcMSH2 from *T. cruzi* I strains displays higher ATPase activity

**Supplementary Fig. 5:**
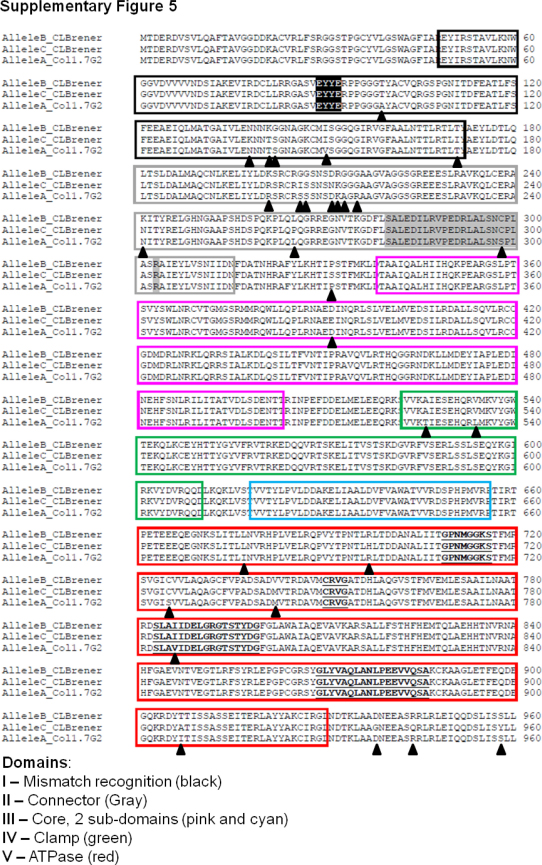
Sequence analysis of TcMSH2 from CL Brener and Col1.7G2 strains. Alignment between TcMSH2 sequences from Col1.7G2 (*T. cruzi* I) and sequences corresponding to alleles B and C from CL Brener (*T. cruzi* II/III hybrid strain). In the N-terminal region, black shading indicates the putative MSH2 mismatch recognition motif, amino acids 89–92. A putative leucine zipper, amino acids 280–300, and a conserved 303 arginine residue are shown by light gray shadings. In the C-terminal region, bolded, underlined amino acids indicate the Walker A motif (P-loop, a Mg^2+^ binding site), amino acids 709–716, the Q-loop (involved in ATP hydrolysis), amino acids 750–754, the Walker B motif (involved in ATP hydrolysis and within the mutS family signature), amino acids 783–799, and the helix-turn-helix motif, amino acids 869–886. The colored blocks represent the different MutS domains: domains I, II, III, IV and V are indicated by black, gray, pink/cyan, green and red boxes, respectively. Black triangles show amino acid substitutions.

To directly investigate the role of TcMSH2 in the differential response of parasite strains to genotoxic treatments, we performed sequence analyses as well as *in vitro* assays with two recombinant isoforms of this protein. Whereas Col1.7G2 is a homozygous *T. cruzi* I strain, presenting two identical *TcMSH2*A alleles, CL Brener is a hybrid cloned strain and presents two *TcMSH2* alleles corresponding to MSH2-haplogroups B or C [18]. The alignment of the complete amino acid sequences of TcMSH2 B and C derived from CL Brener, and the TcMSH2 A from Col1.7G2, identified all common domains and motifs present in MSH2 from other organisms, as described for the *Thermus aquaticus* MutS [16]. A total of 29 amino acid substitutions, concentrated in domains I and II (mismatch recognition domain and connector domain) and domain V (which contains the Walker ATPase motif and the helix-turn-helix motif involved in the protein dimerization), was identified among the three MSH2 haplogroups, 6 of them corresponding to non-conservative substitutions (Supplementary Fig. 5).

**Fig. 3:**
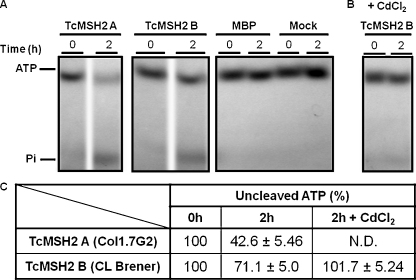
ATPase activity of MSH2 isoforms A (from Col1.7G2) and B (from CL Brener). Affinity purified TcMSH2::MBP proteins were incubated with [γ-^32^P]ATP for 2 h at 37 °C. Negative controls were carried out in the absence of protein or in the presence of MBP only. The position of ATP and inorganic phosphate (Pi) are indicated (A). The effect of cadmium chloride (CdCl_2_) on the ATPase activity of TcMSH2B was evaluated by adding 10 μM CdCl_2_ to the reaction buffer prior to the assay (B) Radioactive bands corresponding to ATP were quantified using the Storm Phosphoimager (GE-HealthCare). ND: not determined (C).

MSH2 are ATPases and, while ATP binding seems not be required for initial recognition of mismatched substrate, ATP hydrolysis is involved in the subsequent steps of MMR [35,36]. Using *in vitro* assays, we compared ATP hydrolysis activities of recombinant TcMSH2 derived from *T. cruzi* I and II strains. As shown in Fig. 3A and C, recombinant TcMSH2 A, the isoform present in *T. cruzi* I strains shows higher levels of ATP hydrolysis after 2 h incubation when compared with the TcMSH2 B isoform, present in *T. cruzi* II strains. In Fig. 3B, it is also shown that cadmium has an inhibitory effect on the *in vitro* TcMSH2 ATPase activity.

### 3.3 *TcMSH2* single knockout clones are more sensitive to oxidative stress

**Supplementary Fig. 6:**
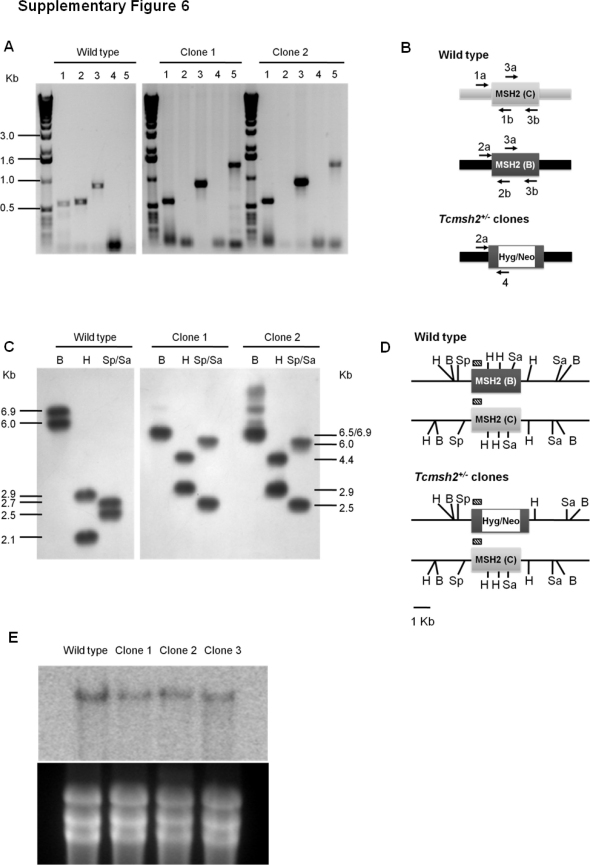
Characterization of *T. cruzi ΔTcmsh2::HYG/TcMSH2* clones. A: agarose gel electrophoresis of PCR products obtained with, lane 1: allele C-specific primers (1a and 1b); lane 2: allele B-specific primers (2a and 2b); lane 3: *Tcmsh2* internal common primers (3a and 3b); lane 4: allele C-specific forward primer and resistance gene-specific reverse primer (1a and 4); lane 5: allele B-specific forward primer and resistance gene-specific reverse primer (2a and 4). In B is shown a schematic diagram of the *TcMSH2 locus* in the CL Brener strain genome (wild type and *ΔTcmsh2::HYG/TcMSH2* clones) and annealing sites of all primers used in A. In C is shown a Southern blot of *T. cruzi* genomic DNA from epimastigote cultures of the CL Brener wild type and *ΔTcmsh2::HYG/TcMSH2* clones. Parasite DNA was digested with *Bam*HI, *Hind*III and *Sph*I/*Sal*I, hybridized with the indicated labelling probe and exposed to a X-Ray film. The sizes of probed fragments are indicated. The probe used in Southern and Northern blots is represented by hachured lines and the restriction sites for *Bam*HI (B), *Hind*III (H), *Sph*I (Sp) and *Sal*I (Sa) are indicated in D. In E is shown the expression of the *TcMSH2* transcripts. Northern blot analyses were performed with total RNA extracted from epimastigotes of CL Brener wild type and *ΔTcmsh2::HYG/TcMSH2* clones. Messenger RNA samples were hybridized with a radiolabed probe indicated in D. The bottom part of the figure shows ethidium bromide staining of rRNA bands to indicate the amount of mRNA loaded on the gel.

To examine genetically the involvement of the MMR pathway in the differential response of *T. cruzi* to DNA damage, we attempted to generate null mutants for the *TcMSH2* gene. Using plasmid constructs aimed at homologous recombination with the *TcMSH2* gene, single allele deletions of *TcMSH2* (*ΔTcmsh2::HYG/TcMSH2* parasites) could readily be achieved in different parasite strains. However after several attempts, including transfections with constructs that are specific for both CL Brener alleles, we were not able to generate *TcMSH2* null mutants. As shown in Supplementary Fig. 6A, DNA amplifications with primers specific for *TcMSH2* alleles B and C, which are present in the CL Brener genome (see Supplementary Fig. 6B) and DNA isolated from two transfected cloned cell lines showed that the resistance gene was integrated at the *TcMSH2-B* allele. Disruption of the *TcMSH2-B* allele was also confirmed by Southern blot (Supplementary Fig. 6C and D) and by the analysis of *TcMSH2* transcript levels, which were reduced to about 50% after the single-allele deletion (Supplementary Fig. 6E). Analysis of more than 10 distinct *ΔTcmsh2::HYG* clones showed that, in every case, deletion of the *TcMSH2-B* allele had occurred (data not shown).

**Fig. 4:**
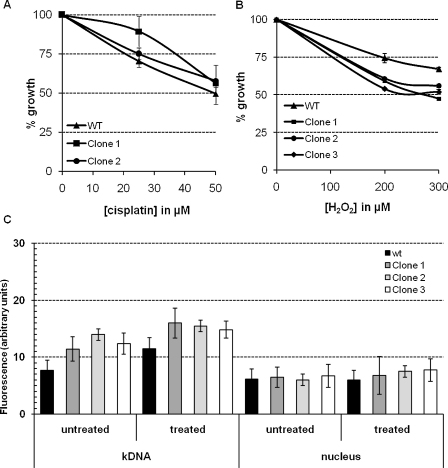
Survival of *ΔTcmsh2::HYG/TcMSH2* clones in the presence of cisplatin and H_2_O_2_ and accumulation of 8-oxoguanine after oxidative stress damage. Wild type and *ΔTcmsh2::HYG/TcMSH2* clones were treated in early log phase with 25 and 50 μM cisplatin (A) or 200 and 300 μM H_2_O_2_ (B). After 5 days, viable cell numbers were determined using erythrosine B dye exclusion. Accumulation of 8-oxoG in the nucleus and kinetoplast DNA in wild type and *ΔTcmsh2::HYG/TcMSH2* mutants was measured before and after treatment with 300 μM H_2_O_2_ (C). Each bar represents the mean ± SD of 100 cells analyzed. Statistical differences (*p* ≤ 0.05) were observed between clone 2 and wt cells in the analysis of kDNA both in untreated and treated parasites.

To investigate the phenotype of *TcMSH2* mutants, epimastigote culture of wild type and *ΔTcmsh2::HYG/TcMSH2* clones were compared. No significant difference of the growth curves of wild type and mutant cells were observed (data not shown). We also compared the growth of wild type and *ΔTcmsh2::HYG/TcMSH2* clones in the presence of increasing concentrations of cisplatin and found no differences (Fig. 4A). In contrast, when treated with 200 or 300 μM H_2_O_2_, all three cloned *ΔTcmsh2::HYG/TcMSH2* mutants presented a significantly lower tolerance (*p* ≤ 0.05) than wild type cells (Fig. 4B). The effect of H_2_O_2_-induced oxidative damage was also observed by measuring the levels of 8-oxoG in the genome of wild type and *ΔTcmsh2::HYG/TcMSH2* cells (Fig. 4C). Although we observed no significant differences in the levels of 8-oxoG present in the nuclei of wild type and mutant strains before or after treatment with H_2_O_2_, increased amounts of the oxidized base were observed in the kDNA of *ΔTcmsh2::HYG/TcMSH2* mutant cell lines compared to wild type parasites (*p* ≤ 0.05).

### 3.4 *Trypanosoma brucei MSH2* mutants display loss of mitochondrial DNA due to oxidative damage

**Fig. 5:**
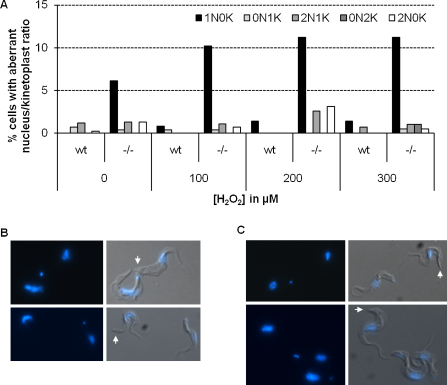
Effect of MSH2 deletion on the DNA content of *T. brucei* before and after hydrogen peroxide exposure. (A) Quantitation of the nuclear (N) and kinetoplast (K) DNA content of *T. brucei* wild type and *ΔTcmsh2::BSD/Tcmsh2::PUR* mutants after 72 h growth in the presence or absence of increasing concentrations of H_2_O_2._ DNA in the cells was visualized by DAPI staining; >500 cells were counted in each sample. Only aberrant cells that differ from the expected N/K ratios (1N1K, 1N2K or 2N2K) are shown, as a percentage of the total population. (B, C) Examples of *ΔTcmsh2::BSD/Tcmsh2::PUR* cells lacking detectable kDNA are shown after 72 h growth in 100 μM H_2_O_2_ (B) or without H_2_O_2_ treatment (C); in all cases the cells are shown both as a DAPI-stain image or as a merge of DAPI and differential interference contrast images and arrows denote the cells lacking kDNA, which are shown beside 1N1K cells for comparison.

In contrast to *T. cruzi*, *MSH2* null mutants have been generated in *T. brucei* and are viable, at least in the bloodstream (mammalian-infective) stage of this parasite [21]. Similar to *T. cruzi ΔTcmsh2::HYG/TcMSH2* clones, *T. brucei MSH2* null mutants (*ΔTbmsh2::BSD/Tbmsh2::PUR*) are more sensitive to H_2_O_2_ than wild type [21]. To ask if this phenotype is also a manifestation of mitochondrial DNA damage, *TbMSH2* null mutants and wild type parasites were grown in increasing concentrations of H_2_O_2_ for 72 h and the DNA content of the cells analyzed microscopically after staining with DAPI. Kinetoplast (K) and nuclear (N) DNA synthesis and segregation occur at distinct times during the *T. brucei* cell cycle [37], meaning that parasites with 1N1K, 1N2K and 2N2K contents are found, normally representing the majority (∼98%) of cell types in wild type cultures; the small minority of cells with differing N–K ratios (‘others’) arise due to errors in DNA replication, segregation or mitosis. Here, we found that, even before H_2_O_2_ treatment, *Tbmsh2* mutants displayed an elevation in the proportion of aberrant cells. Whereas the very small numbers of these aberrant cells in the wild type were essentially equally distributed between 0N1K and 2N1K types (Fig. 5A), virtually all were characterized by a loss of kDNA (predominantly having a 1N0K configuration) in the *Tbmsh2* null mutants (Fig. 5A–C). Treatment of *Tbmsh2* mutants with H_2_O_2_ accentuated this loss of kDNA to an extent that was substantially more pronounced (*p* ≤ 0.05) than in wild type cells (Fig. 5A). These data suggest that in *T. brucei*, MSH2 deficiency, before and after H_2_O_2_-induced damage, results in accelerated loss of mitochondrial DNA, consistent with a function for MSH2 in mitochondrial genome repair or protection.

## 4 Discussion

MMR corrects mismatches generated during DNA replication. It also contributes to the control of endogenous levels of oxidized DNA bases present in the genome such as 8-oxoG [14]. In the *T. cruzi* population, MSH2, a central component of MMR, is encoded by three distinct alleles which can be correlated with the three major *T. cruzi* lineages [17,18]. Because distinct levels of genetic variability have been described among parasite strains belonging to each MSH2 haplogroup, we investigated the activity of this MMR protein and its involvement in the response of different parasite strains to various DNA damaging agents. In so doing, we reveal two things. First, the level of MMR activity is distinct between *T. cruzi* lineage I and II strains. Second, we obtained evidences indicating that MSH2 in both *T. cruzi* and *T. brucei* provides a hitherto unseen function in protecting mitochondrial DNA from oxidative damage.

The involvement of MMR in generating genetic diversity and drug resistance has been described in several organisms, including pathogenic bacteria and other protozoan parasites. Analyses of hypermutable bacteria of different species isolated from human clinical isolates have shown that *mutS* inactivation is implicated in the mutator phenotype [38]. In *Toxoplasma gondii*, disruption of mitochondrial *MSH1* was found in parasites selected for monensin-resistance, a drug proven to be effective against apicomplexan parasites. As expected, the *MSH1* mutants were also resistant to the alkylating agent methylnitrosourea [39]. *Plasmodium* species are the only eukaryotes that encode two *MSH2* homologs. Disruption of one the *MSH2* loci in *Plasmodium falciparum* resulted in a slightly higher frequency of parasites resistant to 5-Fluoroorotate when mosquito-passaged mutants and wild type parasites were compared, suggesting that the function of the two *PfMSH*2 genes may overlap [40]. Here we show that *T. cruzi* I and II strains respond differently when treated with genotoxic agents, such as cisplatin and MNNG. Because this difference was abolished when MMR activity was inhibited by cadmium, we suggest that the differential response among strains to these DNA damaging agents is due to differential activity of one of more components of the MMR. In agreement with previous studies with MMR mutants in different eukaryotic cells [13], the observed increased tolerance to cisplatin and MNNG in *T. cruzi* II parasites can be inferred to result from less efficient MMR. This is also consistent with previous results showing that *T. cruzi* II strains have increased nuclear genetic variability when compared with strains belonging to *T. cruzi* I lineage, an observation that may have major epidemiological significance [6]. The differences observed between strains in the response to genotoxic agents cannot be associated with different levels of expression of *TcMSH2*, since, in all strains analyzed, the levels of *TcMSH2* mRNA and protein were found to be similar. However, because *T. cruzi* I and *T. cruzi* II strains present distinct TcMSH2 isoforms, we proposed that sequence polymorphisms in TcMSH2 may be at least partially responsible for the differences in the response to these DNA damaging agents, an assumption that was corroborated by the results showing differences in ATPase activities from recombinant proteins MSH2A and MSH2B. Notwithstanding, variations in the efficiency of other components of MMR, as well as from other DNA repair pathways are also likely to be involved in modulating mutation rates in the *T. cruzi* population. Attempts to determine whether differences in mutation rates can be observed among distinct strains are underway.

To further investigate the role of TcMSH2 protein, we attempted to generate null mutants for the *TcMSH2* gene. Unexpectedly, we were unable to delete both alleles. This inability to generate *MSH2* null mutants is in sharp contrast with most other eukaryotes, including *T. brucei* bloodstream forms. Bloodstream forms of *TbMSH2* null mutants are viable, present an increased rate of sequence variation of nuclear microsatellite *loci* and increased tolerance to MNNG, as well as an increased frequency of homologous recombination [21,41]. The availability of *TbMSH2* null mutants allowed us to test whether the *TcMSH2* gene can complement MMR deficiency in *T. brucei* [22]. Although we were able to demonstrate heterologous expression of *T. cruzi* MSH2, MMR complementation, as assayed by resistance to MNNG and microsatellite instability, was not achieved. However, expression of *TcMSH2* in *TbMSH2* null mutants resulted in reversion of the sensitivity to H_2_O_2_-induced oxidative stress. The observation described here that this mutation gives rise to a significant percentage of the dyskinetoplastic cells in *T. brucei*, even in the absence of induced oxidative stress, is striking. In contrast to *T. cruzi* epimastigote metabolism, which is largely dependent on oxidative phosphorylation and generates endogenous oxidative damage, *T. brucei* bloodstream forms rely exclusively on glycolysis for ATP production, which may explain why *TbMSH2* null mutants are viable.

Taken together, the results of the complementation assays in *T. brucei*, the non-viability of *TcMSH2* mutants, and the common phenotypes observed in the *TcMSH2* single knockout and *TbMSH2* null mutants suggest that MSH2 provides a function related to mitochondrial genome integrity in addition to its role in nuclear MMR. Such additional functions may be independent of at least some other components of MMR, since *TbMLH1* null mutants do not present increased sensitivity to H_2_O_2_ [22]. This finding is consistent with the observation here that incubation of *T. cruzi* cells with cadmium abolished the differences observed between strains in response to treatment with cisplatin and MNNG, but not with H_2_O_2_:cadmium is known to suppress the ATPase activity of MSH2/MSH6 complexes [33], which is likely to impair interaction with MLH1-containing heterodimers [36]. In both *Trypanosoma* species MSH2 abundance in *MSH2* mutants appears sufficient for nuclear MMR function. However, the *Tcmsh2* mutants showed higher sensitivity to H_2_O_2_-induced oxidative stress than wild type cells and increased accumulation of 8-oxoG, particularly in the kDNA. It can be speculated that the involvement of TcMSH2 in the response to oxidative stress is so relevant to mitochondrial function that a loss of a single allele would have a significant impact*.* The nature of such a mitochondrial role for MSH2 is unknown. Perhaps the protein compensates for the lack of MSH1, a mitochondrial DNA repair protein, for which an orthologous gene could not be identified in the *T. cruzi* genome [4]. A role of MSH2 in the control of oxidative DNA damage has been previously described in other cell types, such as mouse embryo fibroblasts in which increased levels of DNA 8-oxoG were detected after inactivation of the *MSH2* gene [42]. Additional functions of MSH2, MSH3, and PMS2 mismatch repair proteins have also been described, including homologous recombination, anti-recombination, DNA damage signaling, apoptosis, as well as site-specific mutagenesis during immunoglobulin somatic hypermutation and class switch recombination (reviewed in [43]). Nevertheless, these functions have been limited to nuclear activities, and the mitochondrial role we see here for MSH2 appears not to have been described thus far in any other organism.

Being an intracellular parasite, oxidative damage may have a significant effect on mutation rates in *T. cruzi*. In addition of MMR, other DNA repair pathways such as Base Excision Repair (BER) are certainly involved in the parasite response to oxidative damage in DNA. We and others have characterized two DNA polymerase beta from *T. cruzi* and showed that both enzymes co-localize with the parasite kinetoplast [44]. *T. cruzi* has a complex antioxidant defense system that includes enzymes like Fe-SOD (mitochondrial iron-containing superoxide dismutase), TcMPX and TcCPX (mitochondrial and cytoplasmic tryparedoxin peroxidases, respectively), and TcAPX (ascorbate-dependent haemoperoxidase), which are upregulated during transformation of the insect-derived non-infective epimastigotes into the infective metacyclic trypomastigote [45]*.* The evidences presented here indicating a novel role of MSH2 in *T. cruzi* and *T. brucei* in the complex mechanisms responsible for the oxidative stress response require further investigation, particularly regarding the study of MSH2 sub-cellular localization and interactions with other proteins. Whether this new role of MSH2 is part of the driving force behind the differential genetic variability observed within the *T. cruzi* population, it also remains to be investigated.
